# Object-in-Place Associative Recognition Memory Depends on Glutamate Receptor Neurotransmission Within Two Defined Hippocampal-Cortical Circuits: A Critical Role for AMPA and NMDA Receptors in the Hippocampus, Perirhinal, and Prefrontal Cortices

**DOI:** 10.1093/cercor/bht245

**Published:** 2013-09-12

**Authors:** Gareth Robert Issac Barker, Elizabeth Clea Warburton

**Affiliations:** MRC Centre for Synaptic Plasticity, School of Physiology and Pharmacology, University of Bristol, Medical Sciences Building, University Walk, Bristol BS8 1TD, UK

**Keywords:** brain circuits, encoding, glutamate receptors, plasticity, retrieval

## Abstract

Object-in-place associative recognition memory depends on an interaction between the hippocampus (HPC), perirhinal (PRH), and medial prefrontal (mPFC) cortices, yet the contribution of glutamate receptor neurotransmission to these interactions is unknown. NMDA receptors (NMDAR) in the HPC were critical for encoding of object-in-place memory but not for single-item object recognition. Next, a disconnection procedure was used to examine the importance of “concurrent” glutamate neurotransmission in the HPC-mPFC and HPC-PRH. Contralateral unilateral infusions of NBQX (AMPAR antagonist), into the HPC-mPFC, or HPC-PRH, either before acquisition or test, impaired object-in-place performance. Thus, both circuits are necessary for encoding and retrieval. Crossed unilateral AP5 (NMDAR antagonist) infusions into the HPC-mPFC or HPC-PRH impaired encoding, but not retrieval. Specifically crossed HPC-mPFC infusions impaired both short-term (5 min) and longer term (1 h) memory while HPC-PRH infusions impaired longer term memory only. This delay-dependent effect of AP5 in the HPC-PRH on object-in-place memory, accords with its effects in the PRH, on single item object recognition memory, thereby suggesting that a single PRH synaptic plasticity mechanism underpins different recognition memory processes. Further, blocking excitatory neurotransmission in any pair of structures within the networks impaired “both” encoding and retrieval, thus object-in-place memory clearly requires network interdependency across multiple structures.

## Introduction

Object-in-place associative recognition memory requires a subject to make an association between an object and place in which it was previously encountered. In humans, object-in-place memory enables judgments of prior occurrence within an environment and is readily acquired during a single encounter and therefore is essential for normal everyday living. Object-in-place associative recognition memory has been shown to be disrupted in patients with Alzheimer's Disease (Fowler et al. 2002), but interestingly also in patients with schizophrenia (Wood et al. 2002; Burglen et al. 2004), suggesting the involvement of common brain regions such as the HPC, which, in humans, is critical for object-in-place memory (for review, see Owen et al. 1995; Milner et al. 1997) and interconnected cortical regions. Importantly, object-in-place memory may be tested in rats using single-trial spontaneous object memory tasks that do not require reinforcement or lengthy training periods and as such closely parallel tasks that measure human recognition memory (Dere et al. 2007). Indeed, object-in-place memory tasks have been used to explore sex differences on cognitive function in rats (Cost et al. 2012) and cognitive function in animal models of schizophrenia (Howland et al. 2012) drug addiction (Reichel et al. 2012) and mild cognitive impairment (Douma et al. 2011).

In rats, object-in-place memory formation has also been shown to depend critically on the HPC (Bachevalier and Nemanic 2008; Komorowski et al. 2009; Barker and Warburton 2011a) which is both anatomically and functionally interconnected with the mPFC, and PRH (Jay and Witter 1991; Burwell et al. 1995; Gaffan and Parker 1996; Lee and Solivan 2008; Wilson et al. 2008; Jo and Lee 2010; Barker and Warburton 2011b). The HPC has been shown to functionally interact with both the PRH and mPFC during object-in-place memory performance. However, the necessity for multiple hippocampal-cortical circuits was originally demonstrated using permanent lesion techniques which cannot dissociate the underlying plasticity mechanisms involved in memory formation or dissect whether different regions within the circuit are selectively involved in encoding or retrieval. To address such questions, reversible interventions produced by localized pharmacological infusions at distinct stages of the object-in-place task must be used.

Of particular significance to investigations of the neurochemical basis of object-in-place memory is the role of both AMPA receptor (AMPAR) and NMDAR neurotransmission. AMPARs regulate fast excitatory neurotransmission, and AMPAR antagonism in the PRH has been shown to block both encoding and retrieval of object-in-place memory (Barker and Warburton 2008) and single-item object recognition memory (Winters and Bussey 2005), while AMPAR antagonism in the mPFC impaired encoding and retrieval of object-in-place memory only (Barker and Warburton 2008). NMDAR are critical for the induction of synaptic plasticity, including both long-term potentiation and long-term depression (Collingridge et al. 1983; Bear and Malenka 1994; Malenka and Bear 2004; Hunt and Castillo 2012). While the synaptic plasticity mechanisms that underlie the formation of such associative recognition memory have not been established, there is much evidence to support the hypothesis that within the PRH, an long-term depression-like mechanism mediates the storage of single-item object recognition memory (Brown and Bashir 2002; Griffiths et al. 2008; Banks et al. 2012). Interestingly, intra-PRH infusions of AP5, a NMDAR antagonist, impaired object-in-place memory tested following a 1-h retention delay but not a 5-min delay (Barker and Warburton 2008) suggesting that different plasticity mechanisms underlie short-term and long-term recognition memory encoding in the PRH. It is therefore now critical to explore the effects of disruption of both AMPAR and NMDAR neurotransmission within the HPC-PRH-mPFC circuit, and to explore whether common cellular mechanisms within these brain regions, mediate the formation of different types of recognition memory.

The role of the HPC in object recognition has, for a long time, been controversial as a number of lesion studies have shown that damage to this region has little effect on subjects' ability to judge the prior occurrence of single objects (Winters et al. 2004; Forwood et al. 2005; Barker and Warburton 2011a) while other studies reported significant impairments (Clark et al. 2000). Baker and Kim (2002) demonstrated impaired long-term, but not short-term, object recognition memory following intrahippocampal AP5 infusions. Concerning the involvement of hippocampal NMDAR in object-in-place memory, systemic administration of the NMDAR antagonist, CGP-40116, in primates, produced only a mild deficit in both acquisition and retrieval (Gutnikov and Gaffan 1996), while in contrast, the selective deletion of the NR1 subunit of the NMDAR in the CA1 region of the HPC in mice produced a significant impairment (Place et al. 2012). In light of these disparate findings, an evaluation of the role of hippocampal glutamatergic receptor transmission in object recognition and object-in-place memory is now necessary.

The present study addressed important questions concerning the underlying mechanisms of HPC involvement in single-item object recognition and object-in-place memory. Experiment 1 examined whether NMDA-dependent plasticity mechanisms in the HPC are critical for the acquisition or retrieval of object-in-place memory, and aimed to resolve the controversy surrounding the role of hippocampal NMDAR in single-object recognition memory. Experiments 2–5 examined whether the acquisition or retrieval of object-in-place memory requires concurrent glutamate neurotransmission within the hippocampal-cortical circuitry using a disconnection analysis. In examining the importance of NMDAR in recognition memory, we tested the rats following a 5-min delay, to assess shorter term memory or following a 1-h delay to assess longer-term memory.

## Materials and Methods

All experiments were conducted in 250 g male-pigmented rats (DA strain, Bantin and Kingman, Hull, UK), housed in pairs under a 12-h/12-h light/dark cycle (light phase 18:00–6:00 h). Behavioral testing was conducted during the dark phase. Food and water were available ad libitum. All animal procedures were performed in accordance with United Kingdom Animals Scientific Procedures Act (1986) and associated guidelines. All efforts were made to minimize suffering and the number of animals used.

### Surgery

The rats were divided into 3 groups for surgery prior to the start of behavioral testing. Group 1 (HPC, *n* = 12) had bilateral cannulae aimed the HPC, Group 2 (HPC-mPFC, *n* = 12) had bilateral cannulae aimed at the HPC and mPFC, Group 3 (HPC-PRH, *n* = 12) had bilateral cannulae aimed at the HPC and PRH. In Groups 2 and 3, the total number of cannulae implanted in each rat was 4. Each rat was anesthetized with isoflurane (induction 4%, maintenance 2–3%) and secured in a stereotaxic frame with the incisor bar set at 3.3 mm below the interaural line. Stainless steel guide cannulae (26 gauge, Plastics One, Bilaney, UK) were implanted through burr holes in the skull at coordinates relative to bregma (Table 1). In the HPC-PRH group, the cannulae aimed at the HPC were positioned 15° relative to the horizontal plane. All cannulae were anchored to the skull by stainless steel screws (Plastics One, Semat, UK) and dental acrylic. Following surgery, each animal received fluid replacement (5 mL saline, s.c.) and analgesia (0.05 mL Temgesic, i.m.) was housed individually for 1-week postsurgery and then in pairs. The animals were allowed to recover for 14 days before habituation to the apparatus began. Between infusions, 33-gauge obdurators (Plastics One, Semat, UK) kept the cannulae patent.

**Table 1:** Mean exploration times ± SEM in the object-in-place, object recognition, and object location tasks after bilateral preacquisition infusion of AP5 into the HPC

Task	Delay	Group	Exploration in acquisition phase (s)	Exploration in test phase (s)
Object in place	5 min	Vehicle	78.3 ± 6.5	36.8 ± 4.9
		AP5	79.1 ± 8.3	30.2 ± 3.7
	1 h	Vehicle	74.9 ± 6.4	40.3 ± 6.0
		AP5	82.1 ± 7.2	39.3 ± 3.3
	1 h	Vehicle	92.0 ± 7.8	35.3 ± 4.8
		AP5	90.6 ± 7.1	39.2 ± 5.7
Object recognition	1 h	Vehicle	30.1 ± 3.3	34.8 ± 4.4
		AP5	36.9 ± 0.8	28.5 ± 2.8
Object location	1 h	Vehicle	55.0 ± 7.8	34.1 ± 3.2
		AP5	50.9 ± 6.9	41.0 ± 3.2

### Infusion Procedure

NBQX, (Ascent Scientific) a competitive AMPAR receptor antagonist, and AP5 (Ascent Scientific) a competitive NMDAR antagonist were dissolved in sterile 0.9% saline solution and infused at 1 and 25 mM/side, respectively. The infusions were made through a 33-gauge infusion needle (Plastics One, Bilaney, UK) inserted into the implanted cannulae and attached to a 25-µL Hamilton syringe via polyethylene tubing. For infusions into the HPC, a volume of 0.5 µL was injected, over a 2-min period by infusion pump (Harvard Bioscience, Holliston, MA, USA). For infusions into the PRH and mPFC, a volume of 1.0 µL per hemisphere was injected over 2 min. The volumes used have been used extensively previously (Winters and Bussey 2005; Akirav and Maroun 2006; Barker and Warburton 2008) and have been shown to achieve a drug spread of 1–1.5 mm^3^ (Martin 1991; Attwell et al. 2001). Following the infusion, the needle remained in place for a further 5 min.

### Histology

At the completion of the study each rat was anesthetized with Euthetal (Rhône Mérieux) and perfused transcardially with phosphate buffered saline followed by 4% paraformaldehyde. Following removal, the brain was postfixed in paraformaldehyde for a minimum of 2 h then transferred to 30% sucrose in 0.2 M phosphate buffer for 48 h. Coronal sections (50 µm) were cut on a cryostat and stained with cresyl violet. Cannulae locations were checked against a rat brain atlas (Swanson 1998).

To determine the extent of drug spread, flurophore-conjugated muscimol (Invitrogen, UK) was diluted to a concentration 0.5 mg/mL (5% DMSO/saline) and infused into the brain regions of interest. Anterior–posterior (AP) drug spread was calculated measuring the extent of fluorescence across serial sections. Medial-lateral (ML) and dorsal-ventral (DV) spread was measured using imaging program, IMAGEJ (National Institutes of Health, USA).

### Apparatus

Investigation of the objects occurred in a wooden open-topped arena (90 × 100 cm), the walls (height 50 cm) of which were painted gray and were surrounded by a black cloth (height 1.5 m). The floor was covered with sawdust. An overhead camera and a DVD recorder monitored and recorded the animal's behavior, which was scored manually on-line. The stimuli were copies of objects composed of “Duplo” (Lego UK Ltd, Slough, UK) that varied in shape, color and size (9 × 8 × 7 cm to 25 × 15 × 10 cm) and could not be displaced.

### Behavioral Testing

#### Pre-Training

All animals were handled for a week then habituated to the empty arena for 10–15 min daily for 4 days.

#### Object-in-Place Task

**Figure 1.**
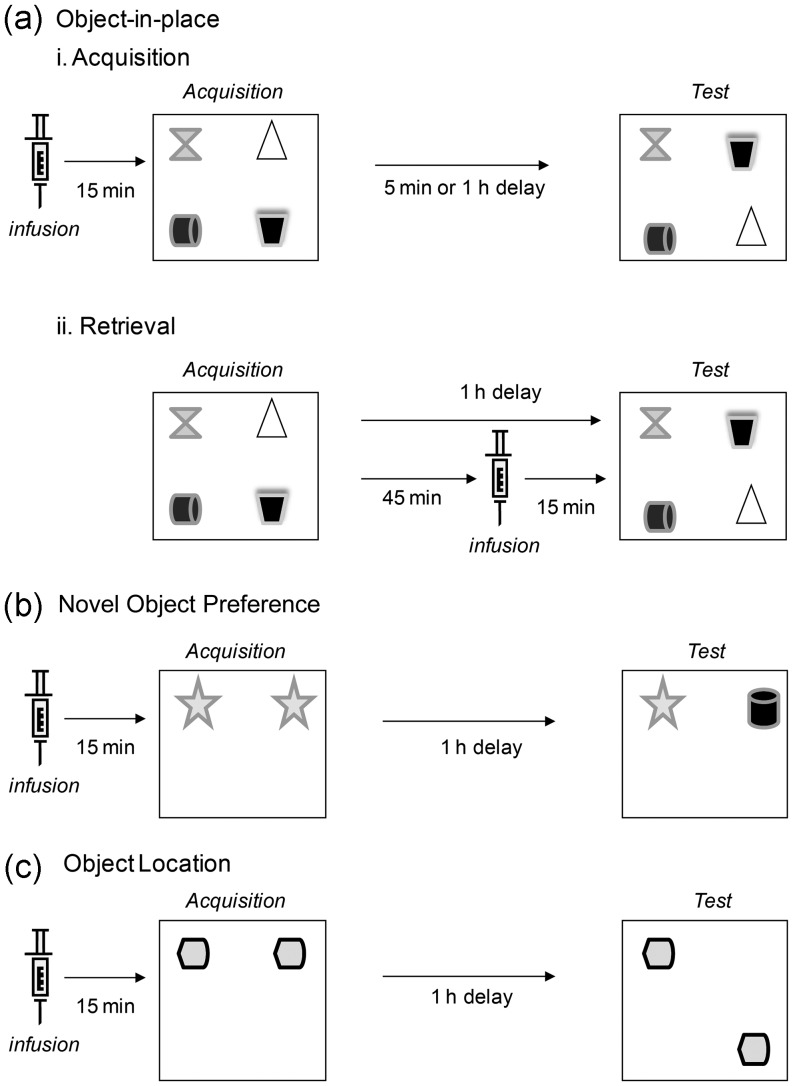
Diagram of the 3 object recognition memory tasks and the delays used. (*a*) object-in-place task (i) to assess drug effects on encoding, the infusion was give 15 min before the start of the acquisition phase, and (ii) to assess drug effects on retrieval, the infusion was given 15 min before the test phase; (*b*) novel object preference task; (*c*) object location task,

The task comprised an acquisition and a test phase separated by a 5-min or a 1-h delay (Fig. 1*a*). In the acquisition phase, each subject was placed in the center of the arena which contained 4 different objects in the corners 15 cm from the walls, and allowed to investigate the objects for 5 min before being removed and placed in the home cage for the delay. During the delay, all objects were cleaned with alcohol. In the test phase (duration 3 min), each subject was replaced in the arena, in which 2 of the objects had exchanged position, and was allowed to investigate the objects. The time spent investigating the objects in the changed position was compared with the time spent investigating the objects in the same position. Object position was counterbalanced between rats. Intact object-in-place memory occurs when the subject spends more time investigating the 2 objects in different locations, compared with the 2 objects in the same locations.

#### Novel Object Preference Task

The acquisition and test phases were separated by a 1-h delay (Fig. 1*b*). In the acquisition phase, duplicate objects were placed near 2 corners in the arena. Each subject was allowed a total of either 40 s of object investigation or 4 min in the arena. At test (duration 3 min), the animal was replaced in the arena, presented with objects in the same positions as at acquisition: one object was the third copy of the object used at acquisition and the other a novel object. Object positions and the objects used as novel or familiar were counterbalanced. If novel object recognition memory is intact subjects spend more time investigating the novel object.

#### Object Location Task

The acquisition and test phases were separated by a 1-h delay (Fig. 1*c*). In the acquisition phase, the rat was allowed to investigate duplicate objects for 3 min. At test (3 min), one object was placed in the same position it had occupied at acquisition while an identical object was placed in the corner diagonally opposite. The position of the moved object was counterbalanced between rats. Intact object location memory is evidenced by subjects spending more time investigating the object in the new location.

### Design

Group 1 (HPC) received bilateral infusions into the HPC only; Groups 2 (HPC-mPFC) and 3 (HPC-PRH) received combined unilateral infusions into the HPC and mPFC or HPC and PRH. The requirement for simultaneous neural transmission in the circuits was assessed by infusing antagonists unilaterally into the HPC and either the mPFC or PRH in either the same hemisphere (HPC-mPFC ipsi/ HPC-PRH ispi) or in opposite hemispheres (HPC-mPFC contra/HPC-PRH contra). To assess drug effects on encoding, drug or vehicle (veh) infusions were made 15 min before acquisition, and memory was tested following a 5-min or a 1-h delay. To assess drug effects on retrieval, infusions were made 15 min before test and memory tested following 1-h delay.

All experiments were run using a cross-over design and each animal re-tested following a 48-h rest period. Thus, there were 12 animals in the ipsi and contra groups, with each animal serving as its own control. Cannula blockage resulted in the occasional loss of an animal (indicated by reduced degrees of freedom in the quoted statistical tests).

### Data Analysis

The experimenter was blind to the infusion status of each animal. The duration of the investigatory behavior (in seconds) of the novel (*t*_nov_) or familiar object (*t*_fam_) was defined as the animal directing its nose toward the object at a distance of <2 cm. Other behaviors, for example, sitting on or resting against the object were not scored. Discrimination between the objects was calculated as follows: (*t*_nov_ – *t*_fam_*)*/(*t*_nov_ + *t*_fam_). This discrimination ratio thus takes into account individual differences in the total object investigation (Ennaceur and Delacour 1988). Statistical comparisons were made using a multifactor analysis of variance (ANOVA; SPSS version 18, IBM). In Experiment 1, the within-subjects factor was treatment (veh vs. drug) and the between-subjects factor was delay (5 min or 1 h). In Experiments 2–5, the within-subjects factor was group (ipsi vs. contra) and in the tests of acquisition, the between-subjects factor was delay (5 min vs. 1 h). Post hoc simple main effects tests were conducted where appropriate and whether individual groups discriminated between the objects was assessed using a within-subject *t*-test (2-tailed). All statistical analyses used a significance level of 0.05.

## Results

### Histology

**Figure 2.**
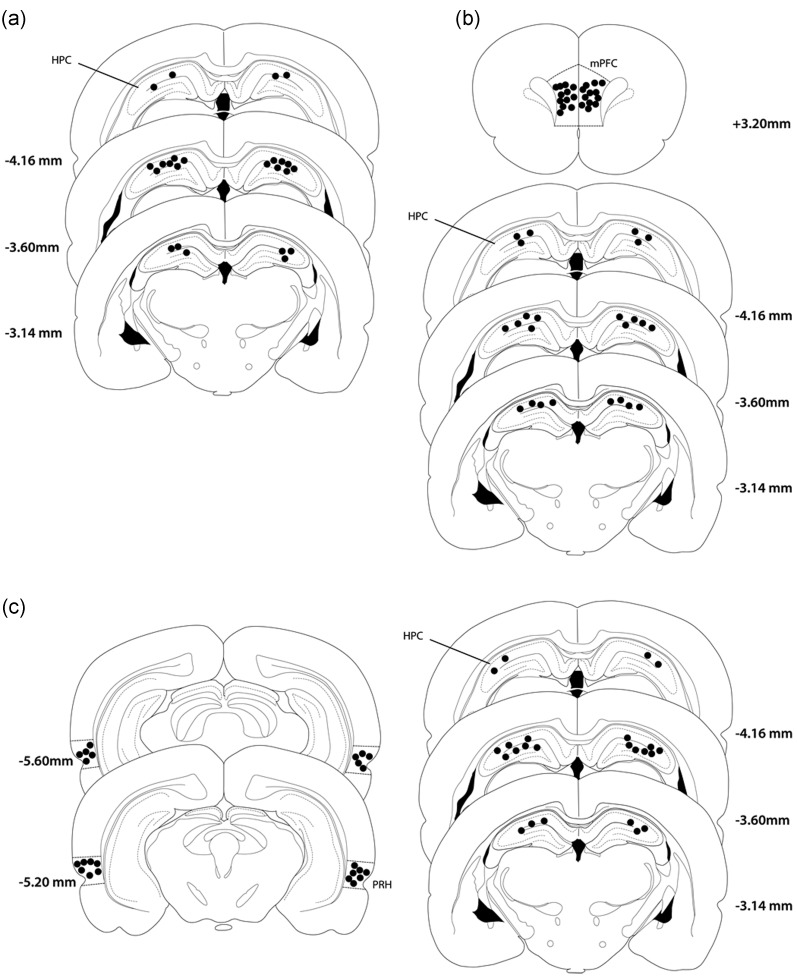
Diagrammatic representation of the individual infusion sites in each animal (*a*) Bilateral HPC group; (*b*)HPC-mPFC group; (*c*) HPC-PRH group. All infusion sites were within the HPC, mPFC, or PRH.

Histological examination confirmed that the tips of the cannulae located in the HPC were located between the CA1 and CA3 subfields. All rats with cannulae implanted in the mPFC had needle tips located in the ventral portion of prelimbic and dorsal portion of the infralimbic regions, and all rats with cannulae implanted in the PRH had the tip of the bilateral cannulae in the PRH (Fig. 2).

Intraperirhinal infusions of flurophore-conjugated muscimol resulted in drug spread restricted to the PRH. Staining extended ∼2.2 mm AP, 1.7 mm DV, and 1.2 mm ML. Infusions into the mPFC revealed drug spread which extended ∼2.45 mm AP, 1.5 DV, and 1.5 mm ML. Intrahippocampal infusions revealed drug spread of 1.62 mm AP, 0.89 mm DV, and 0.89 mm ML.

### Experiment 1

#### NMDA Receptor Neurotransmission Within the HPC is Critical for the Acquisition But Not Retrieval of Object-in-Place Memory

**Figure 3.**
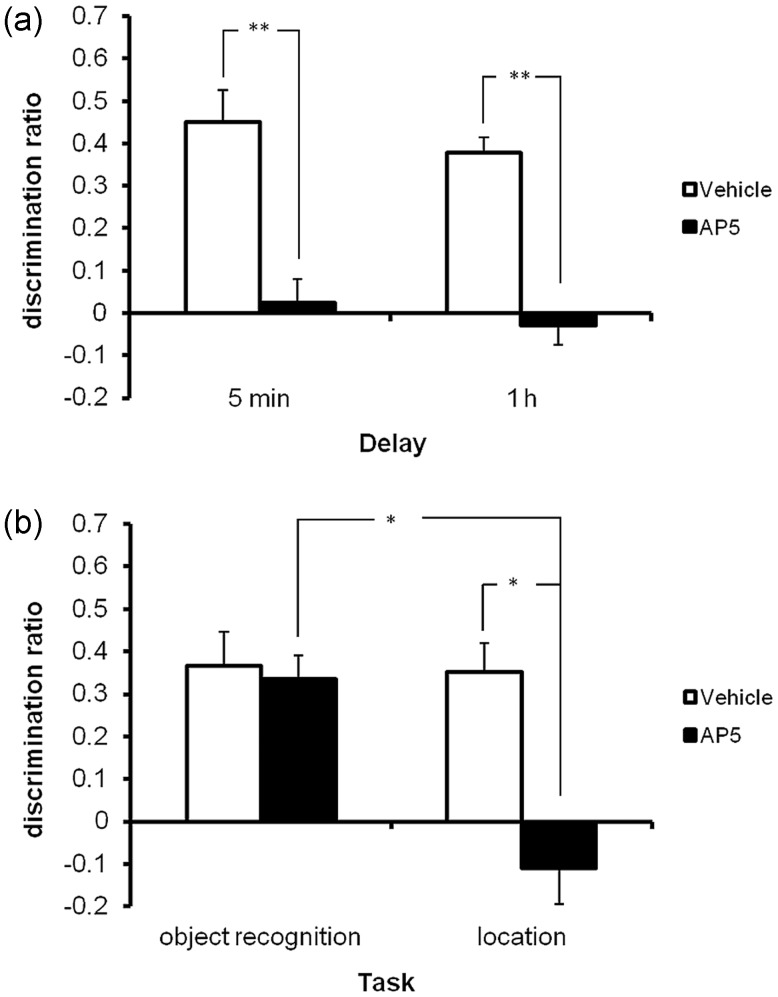
Experiment 1 Bilateral infusions of AP5 into the hippocampus selectively impaired recognition memory depending on the task. Illustrated for each group is the mean (+SEM) discrimination ratio. *P < 0.05, **P < 0.01 difference between groups. (*a*) AP5 infusions into the HPC before the acquisition phase significantly impaired object-in-place performance after a 5-min or 1-h delay. Analyses also revealed that the vehicle-treated animals discriminated between the rearranged and nonrearranged objects in all conditions (5 min *t*_(6)_ = 5.51, *P* < 0.01; 1 h *t*_(6)_ = 9.16, *P* < 0.001) while the AP5 animals did not (5 min *t*_(6)_ = 0.42, *P* > 0.1; 1 h *t*_(6)_ = 0.56, *P* > 0.1). (*b*) AP5 infusions into the HPC before the acquisition phase had no effect on novel object recognition, task but significantly impaired object location memory.

Bilateral administration of AP5 into the HPC before acquisition, so as to be active during memory encoding, impaired object-in-place performance independent of delay (Fig. 3*a*) confirmed by a significant treatment effect (*F*_1,12_ = 46.49, *P* < 0.001), but no significant treatment by delay interaction (*F*_1,12_ = <1.0, *P* > 0.1). Administration of AP5 into the HPC before the test phase had no effect on object-in-place performance data not shown (discrimination ratio: veh 0.34 ± 0.09; AP5 0.36 ± 0.09, *F* < 1.0, *P* > 0.1). Finally, AP5 administration had no effect on overall object investigation levels in the acquisition (treatment by delay *F*_1,12_ = 0.65, *P* > 0.1; treatment *F*_1,12_ = 1.04, *P* > 0.1 or test phases (*F*_1,5_ = 1.02, *P* > 0.1; Table 1).

#### NMDA Receptor Neurotransmission Within the HPC is Required for Acquisition of Object Location But Not Object Recognition Memory

Bilateral administration of AP5 into the HPC before acquisition significantly impaired object location memory but not object recognition memory (Fig. 3*b*). ANOVA revealed a significant treatment effect (*F*_1,12_ = 7.24, *P* < 0.05), a significant treatment by task interaction (*F*_1,12_ = 5.50, *P* < 0.05), and a significant task effect (*F*_1,12_ = 13.69, *P* < 0.01). Further, the AP5-treated animals were significantly worse than the vehicle-treated animals in the location memory task (*P* < 0.05) but not in the object recognition task (*P* > 0.05). There was no effect of AP5 on object investigation levels in acquisition phase of either task, (treatment by task interaction *F*_1,12_ = 0.84, *P* > 0.1) or in the test phase *F*_1,12_ *F*_1,12_ = 4.67, *P* > 0.05; Table 1).

### Experiment 2

#### The HPC-mPFC Circuit is Required for the Acquisition and Retrieval of Object-in-Place Memory, But Not for Object Location or Object Recognition Memory

**Figure 4.**
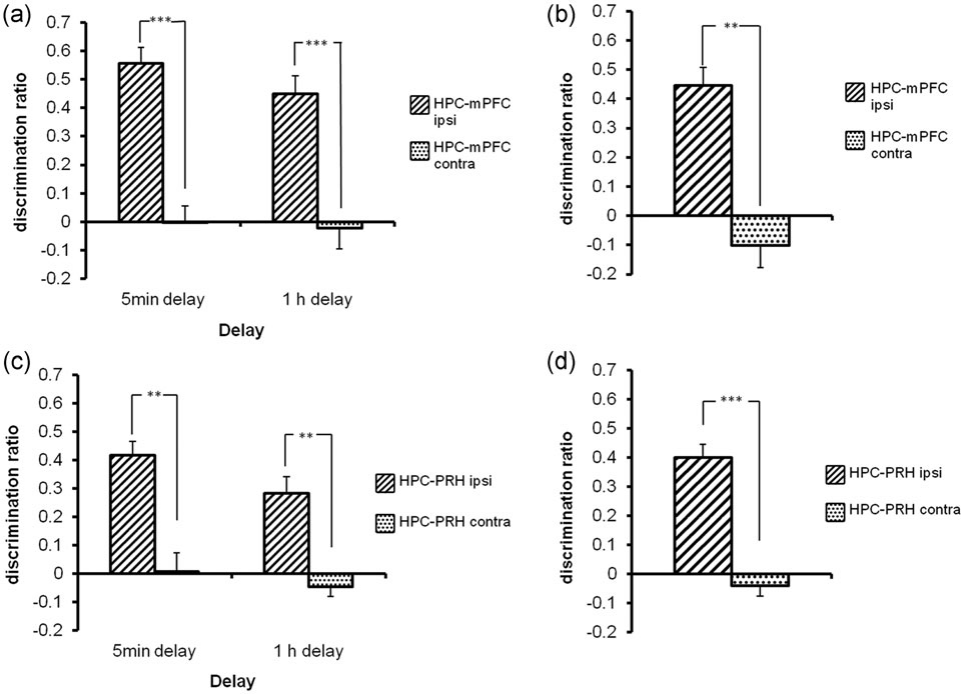
Experiments 2 and 3 NBQX infusion into the HPC-mPFC or HPC-PRH circuits impaired both acquisition and retrieval of object-in-place performance. Illustrated for each group is the mean (+SEM) discrimination ratio. **P < 0.01, ***P < 0.001 difference between groups. (*a*) Encoding unilateral infusion of NBQX into the HPC-mPFC in opposite hemispheres (HPC-mPFC contra) prior to the acquisition phase, impaired object-in-place performance after a 5-min or a 1-h delay while infusions into the same hemisphere (HPC-mPFC ipsi) had no effect. At both delays, the HPC-mPFC ipsi group showed significant discrimination (5 min delay *t*_(9)_ = 9.29, *P* < 0.001; 1 h delay *t*_(8)_ = 6.68, *P* < 0.001) while the HPC-mPFC contra group did not (5 min *t*_(9)_ = −0.01, *P* > 0.1; 1 h *t*_(8)_ = −0.28, *P* > 0.1). (*b*) Retrieval unilateral infusion of NBQX into the HPC-mPFC in opposite hemispheres (HPC-mPFC contra), but not into the same hemisphere (HPC-mPFC ipsi), prior to the test phase, impaired object-in-place performance. The HPC-mPFC ipsi group significantly discriminated between the rearranged and nonrearranged objects in all conditions (*t*_(9)_ = 6.79, *P* < 0.001) while the HPC-mPFC contra animals did not (*t*_(9)_ = −1.24, *P* > 0.1). (*c*) Encoding unilateral infusion of NBQX into the HPC-PRH in opposite hemispheres (HPC-PRH contra) prior to the acquisition phase, impaired object-in-place performance after a 5-min or 1-h delay while infusions into the same hemisphere (HPC-PRH ipsi) had no effect. The HPC-PRH ipsi group showed significant discrimination at both delays (5 min: *t*_(8)_ = 7.92, *P* < 0.001; 1 h: *t*_(8)_ = 3.82, *P* < 0.01) while the HPC-PRH contra group did not (5 min: *t*_(8)_ = −0.08, *P* > 0.1; 1 h: *t*_(8)_ = −1.12, *P* > 0.1). (*d*) Retrieval unilateral infusion of NBQX into the HPC-PRH in opposite hemispheres (HPC-PRH contra) prior to the test phase, but not into the same hemisphere (HPC-PRH ipsi), impaired object-in-place performance. The HPC-PRH ipsi group significantly discriminated between the rearranged and nonrearranged objects (*t*_(8)_ = 7.20, *P* < 0.001); while the HPC-PRH contra did not (*t*_(8)_ = −0.86, *P* > 0.1).

Administration of NBQX before acquisition significantly impaired performance of the HPC-mPFC contra group independent of delay (Fig. 4*a*). There was a significant group effect (*F*_1,17_ = 49.04, *P* < 0.001) but no group by delay interaction (*F*_1,17_ = <1.0, *P* > 0.1). Post hoc analyses confirmed that the HPC-mPFC contra group performed significantly worse than the HPC-mPFC ipsi group at both delays (5 min *P* < 0.001; 1 h *P* < 0.01). There was no significant difference in overall object investigation levels in the acquisition phase (group by delay interaction *F*_1,17_ = 0.07; Table 2).

**Table 2:** Mean exploration times ± SEM in the object-in-place task after unilateral infusion of NBQX into either the HPC and mPFC or the HPC and PRH

Site of infusion	Infusion timing	Delay	Group	Exploration in acquisition phase (s)	Exploration in test phase (s)
HPC-mPFC	Before acquisition phase	5 min	ipsi	58.6 ± 3.6	25.9 ± 2.7
			contra	65.4 ± 4.1	25.0 ± 2.5
		1 h	ipsi	76.1 ± 4.7	31.5 ± 4.4
			contra	85.2 ± 3.9	35.8 ± 3.6
	Before test phase	1 h	ipsi	74.0 ± 4.1	36.5 ± 4.1
			contra	68.7 ± 3.9	38.1 ± 4.5
HPC-PRH	Before acquisition phase	5 min	ipsi	69.6 ± 3.7	24.1 ± 2.3
			contra	67.7 ± 3.1	30.0 ± 2.6
		1 h	ipsi	65.1 ± 6.5	35.2 ± 2.9
			contra	63.8 ± 4.3	30.8 ± 2.6
	Before test phase	1 h	ipsi	68.2 ± 4.5	36.0 ± 3.7
			contra	63.0 ± 6.3	38.4 ± 2.1

Administration of NBQX before test, so as to be active during retrieval, significantly impaired memory in the HPC-mPFC contra group (*F*_1,9_ = 19.64, *P* < 0.01) (Fig. 4*b*). NBQX prior to the test phase produced no significant difference in the amount of test phase object investigation in either group (*F*_1,9_ = 0.20 *P* > 0.1, Table 2).

Preacquisition administration of NBQX into the HPC-mPFC in either the same or opposite hemispheres had no effect on object recognition or object location memory (treatment by task interaction *F*_1,16_ = 0.75, *P* > 0.1) (Supplementary Fig. 1*a*). Further analyses confirmed that NBQX had no effect on the animals ability to discriminate between the novel and familiar objects [HPC-mPFC ipsi *t*_(8)_ = 15.59, *P* < 0.001; HPC-mPFC contra *t*_(8)_ = 2.68, *P* < 0.05] or between the objects in the novel or familiar location [HPC-mPFC ipsi *t*_(8)_ = 5.30, *P* < 0.01; HPC-mPFC contra *t*_(8)_ = 8.83, *P* < 0.01].

### Experiment 3

#### The HPC-PRH Circuit is Required for Both the Acquisition and Retrieval of Object-in-Place Memory, But Not for Object Location or Object Recognition Memory

NBQX administration before acquisition significantly impaired the HPC-PRH contra group independent of delay as shown by the significant group effect (*F*_1,16_ = 39.26, *P* < 0.001), but no group by delay interaction (*F* < 1.0, *P* > 0.1). Post hoc analyses showed that the HPC-PRH contra group performed significantly worse than the HPC-PRH ipsi group at both delays (5 min and 1 h *P* < 0.01) (Fig. 4*c*). There was no effect of NBQX on the overall object investigation levels in the acquisition phase (group by delay interaction *F*_1,16_ = 0.01, *P* > 0.1, Table 2).

NBQX administration before test significantly impaired performance of the HPC-PRH contra group compared with the HPC-PRH ipsi group (*F*_1,8_ = 75.81, *P* < 0.001) (Fig. 4*d*). There was no effect of NBQX on the test phase object investigation levels (*F*_1,8_ = 0.28, *P* > 0.1, Table 2).

Preacquisition administration of NBQX into the HPC-PRH in either the same or opposite hemispheres had no effect on object recognition or object location memory (treatment by task interaction *F*_1,15_ = 1.00, *P* > 0.1) (Supplementary Fig. 1*b*) Further analyses confirmed that NBQX had no effect on the animals ability to discriminate between the novel and familiar objects [HPC-PRH ipsi *t*_(7)_ = 5.45, *P* < 0.01; HPC-PRH contra *t*_(7)_ = 5.30, *P* < 0.01] or between the objects in the novel or familiar location [HPC-PRH ipsi *t*_(8)_ = 6.10, *P* < 0.001; HPC-PRH contra *t*_(8)_ = 7.68, *P* < 0.001].

### Experiment 4

#### NMDA Receptor Neurotransmission Within the HPC-mPFC Circuit is Critical for the Acquisition, But Not Retrieval of Object-in-Place Memory

**Figure 5.**
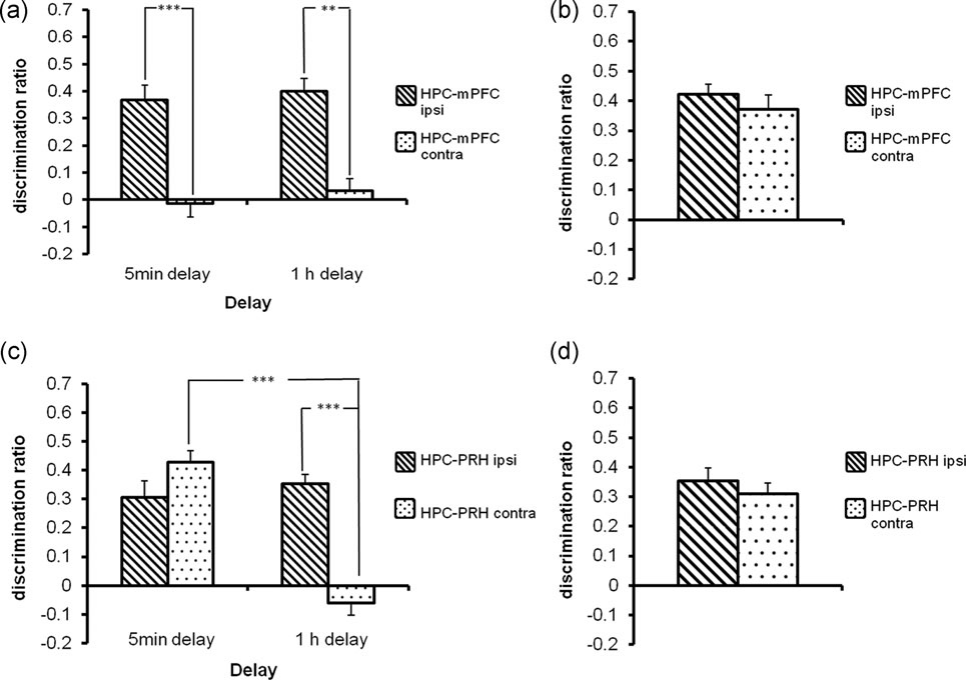
Experiments 4 and 5 AP5 infusion into the HPC-mPFC or HPC-PRH circuits impaired acquisition of the object-in-place task in a delay-dependent manner, but was without effect on retrieval. Illustrated for each group is the mean (+SEM) discrimination ratio. **P < 0.01, ***P < 0.001 difference between groups. (*a*) Encoding unilateral infusion of AP5 into the HPC-mPFC in opposite hemispheres (HPC-mPFC contra) prior to acquisition impaired object-in-place performance following a 5-min or 1-h delay. The ipsi group significantly discriminated between the rearranged and nonrearranged objects (5 min delay *t*_(11)_ = 5.60, *P* < 0.001; 1 h delay *t*_(11)_ = 6.65, *P* < 0.001); while the contra group did not (5 min delay *t*_(11)_ = −0.01, *P* > 0.1; 1 h delay *t*_(11)_ = 0.74, *P* > 0.1). (*b*) Retrieval unilateral infusion of AP5 into the HPC-mPFC in either the same or opposite hemispheres prior to test had no effect on retrieval of object-in-place memory in either group. Both groups significantly discriminated between the rearranged and nonrearranged objects (ipsi *t*_(11)_ = 12.03; contra *t*_(11)_ = 7.06, *P* < 0.001). (*c*) Encoding unilateral infusion of AP5 into the HPC-PRH in opposite hemispheres (HPC-PRH contra) prior to the acquisition phase, impaired object-in-place performance following a 1-h delay, but not a 5-min delay. The HPC-PRH ipsi group significantly discriminated between the rearranged and nonrearranged objects at both delays (5 min *t*_(11)_ = 5.07, *P* < 0.001; 1 h *t*_(10)_ = 11.05, *P* < 0.001) while the HPC-mPFC contra group significantly discriminated at the 5-min delay only (5 min *t*_(11)_ = 10.30, *P* < 0.001, 1-h delay (*t*_(10)_ = −1.32, *P* > 0.1). (*d*) Retrieval unilateral infusion of AP5 into the HPC-PRH in either the same or opposite hemispheres prior to test had no effect on retrieval of object-in-place memory in either group. Both groups significantly discriminated between the rearranged and nonrearranged objects (HPC-PRH ipsi (*t*_(8)_ = 7.60, *P* < 0.001); HPC-PRH contra (*t*_(8)_ = 7.70, *P* < 0.001)).

Simultaneous unilateral AP5 administration into the HPC and mPFC before acquisition significantly impaired object-in-place memory in the HPC-mPFC contra group independent of delay (Fig. 5*a*) as shown by the significant group effect (*F*_1,22_ = 45.03, *P* < 0.001), but no significant group by delay interaction (*F*_1,22_ = <1.0, *P* > 0.1). Post hoc analysis showed that the HPC-mPFC contra group performed significantly worse than the HPC-mPFC ipsi group at both delays (5 min *P* < 0.001; 1 h *P* < 0.01). Ipsilateral or contralateral unilateral infusions of AP5 immediately prior to the test phase, produced no difference in performance between the 2 groups (*F*_1,11_ = <1.0, *P* > 0.1) and neither group was significantly impaired (Fig. 5*b*). There was no effect of AP5 on the amount of overall object investigation completed in the acquisition (group by delay interaction *F*_1,22_ = 0.12, *P* > 0.1 Table 3) or test phases (group by delay interaction *F*_1,22_ = 0.17, *P* > 0.1; Table 3).

**Table 3:** Mean exploration times ± SEM in the object-in-place task after unilateral infusion of AP5 into either the HPC and mPFC or the HPC and PRH

Site of infusion	Infusion timing	Delay	Group	Exploration in acquisition phase (s)	Exploration in test phase (s)
HPC-mPFC	Before acquisition phase	5 min	Ipsi	73.3 ± 5.9	30.0 ± 2.2
			Contra	76.5 ± 4.7	29.9 ± 2.2
		1 h	Ipsi	76.5 ± 5.3	36.3 ± 3.2
			contra	79.0 ± 3.9	39.8 ± 2.2
	Before test phase	1 h	ipsi	84.8 ± 4.2	45.5 ± 3.1
			contra	81.6 ± 4.9	44.7 ± 3.9
HPC-PRH	Before acquisition phase	5 min	ipsi	66.8 ± 5.8	27.0 ± 2.6
			contra	78.3 ± 7.1	27.2 ± 3.2
		1 h	ipsi	80.5 ± 4.9	33.0 ± 5.1
			contra	73.7 ± 5.2	34.3 ± 2.1
	Before test phase	1 h	ipsi	67.9 ± 4.1	42.4 ± 3.2
			contra	66.0 ± 4.6	35.6 ± 2.9

Preacquisition administration of AP5 in the HPC-mPFC ipsi or contra groups had no effect on object recognition or object location memory (treatment by task interaction *F*_1,17_ = 2.40, *P* > 0.1) (Supplementary Fig. 1*c*). Further analyses confirmed that NBQX had no effect on the animals ability to discriminate between the novel and familiar objects [HPC-mPFC ipsi *t*_(10)_ = 5.74, *P* < 0.001; HPC-mPFC contra *t*_(10)_ = 10.37, *P* < 0.001] or between the objects in the novel or familiar location [HPC-mPFC ipsi *t*_(7)_ = 6.40, *P* < 0.001; HPC-mPFC contra *t*_(7)_ = 3.17, *P* < 0.05].

### Experiment 5

#### NMDA Receptor Neurotransmission Within the HPC-PRH Circuit is Critical for the Acquisition, But Not the Retrieval of Object-in-Place Memory

AP5 significantly impaired object-in-place memory in the HPC-PRH contra group in a delay-dependent manner (Fig. 5*c*). ANOVA revealed a significant group by delay interaction (*F*_1,21_ = 34.13, *P* < 0.001), a significant treatment effect (*F*_1,21_ = 10.13, *P* < 0.01), and delay effect (*F*_1,21_ = 21.45, *P* < 0.001). Post hoc analyses revealed that the HPC-PRH contra group were significantly worse than the HPC-PRH ipsi group at 1 h (*P* < 0.001) but not at 5 min (*P* > 0.1). Unilateral infusions of AP5 immediately prior to the test phase, produced no difference in performance between the ipsi and contra groups (*F*_1,8_ = 0.38, *P* > 0.1) and neither group was significantly impaired (Fig. 5*d*). AP5 infusions had no effect on levels of object investigation in the acquisition (group by delay *F*_1,21_ = 3.53, *P* > 0.05) or test phase (group by delay *F*_1,21_ = 0.03, *P* > 0.1; Table 3).

Preacquisition administration of AP5 in the HPC-PRH ipsi or contra groups had no effect on object recognition or object location memory (treatment by task interaction (*F*_1,19_ = 0.11, *P* > 0.1) (Supplementary Fig. 1*d*). Further analyses confirmed that AP5 had no effect on the animals ability to discriminate between the novel and familiar objects [HPC-PRH ipsi *t*_(9)_ = 5.83, *P* < 0.001; HPC-PRH contra *t*_(9)_ = 4.80, *P* < 0.001] or between the objects in the novel or familiar location [HPC-PRH ipsi *t*_(10)_ = 4.54, *P* < 0.001; HPC-PRH contra *t*_(10)_ = 5.71, *P* < 0.001].

## Discussion

This study demonstrated that hippocampal NMDAR are critically involved in the encoding of object-in-place and object location but not object recognition memory. Second, both encoding and retrieval of object-in-place memory were found to depend on 2 hippocampal-cortical circuits, involving the HPC, mPFC, and PRH. Third, it was shown that encoding, but not retrieval of object-in-place memory within these circuits relies upon an NMDAR-dependent mechanism. Significantly, however, the requirement for NMDAR within the HPC-PRH circuit was dependent on the length of the retention delay.

That NMDAR blockade in the HPC impaired the object location and object-in-place tasks but not the novel object preference task demonstrates clear dissociation between the effects of hippocampal manipulations in a spatial and object version of a recognition memory task. As this dissociation was obtained using identical stimulus types, arena, and identical retention delays of 1 h, it cannot be accounted for by differences in the response requirements of the procedures or attentional or motivational processes. The results, therefore, strongly support the position that the HPC is not required for single-object recognition yet is required for the rapid encoding and for the retrieval of single-trial spontaneous object-in-place memory. This finding is consistent with current theoretical models in which the HPC plays a central role in integrating object and spatial information or in representing the relationship between objects and their location (Eichenbaum 2004; Bast et al. 2005) but does not mediate the formation of object familiarity (Brown and Aggleton 2001). The present results also emphasize that encoding, but not retrieval of single-trial object-in-place memory relies upon a NMDA-dependent synaptic plasticity mechanism within the HPC. The drug infusions are likely to result in sustained NMDAR antagonism during early consolidation, which may be of consideration in interpreting the current results, as studies have shown that NMDAR blockade in the PRH at this stage of processing impairs object recognition (Winters and Bussey 2005), although other studies have shown that such infusions into the HPC do not produce mnemonic impairments (Schenberg and Oliveira 2008). The lack of effect of NMDAR on retrieval in the present study is consistent with results from previous object and spatial memory tasks conducted in rats (Day et al. 2003; Winters and Bussey 2005; Barker and Warburton 2008) although it contrasts with the findings of Gutnikov and Gaffan (1996) who showed only a weak involvement of NMDAR in object-place memory in rhesus monkeys, across all stages of memory processing. These differences in the effects of NMDAR antagonism on retrieval could be partly explained by differences between species used, or route of drug administration, that is, local compared with systemic administration.

### Importance of Interactions Between the HPC and Cortical Regions

Crossed HPC-PRH and HPC-mPFC NBQX infusions before the sample and test phases significantly impaired object-in-place performance, thus both neural circuits were engaged in encoding and retrieval. This result is consistent with the view that retrieval depends on activation of the same neural circuits, and indeed the same individual neurons involved in the encoding of the original information (Morris et al. 2003; Eichenbaum 2004; Liu et al. 2012; Tayler et al. 2013). The results also demonstrated that the encoding of object-in-place memory involves simultaneous NMDAR-dependent synaptic plasticity in a number of neural regions within multiple neural circuits.

Crucially, the amnestic effects of NMDAR blockade in the HPC-PRH circuit, but not in the HPC-mPFC circuit, were found to be delay dependent, that is, a memory impairment following a long, but not a short delay. This is an important finding as it provides a hypothesis concerning the role of the PRH in object-in-place associative recognition memory. The PRH has been shown to be crucial for both object familiarity discrimination and object identification (Murray and Richmond 2001; Buckley 2005; Bussey and Saksida 2005), and while both processes have been shown to be blocked by AP5 (Winters et al. 2010), the effect of NMDAR antagonism, in the PRH, on familiarity discrimination is also delay-dependent (Winters and Bussey 2005; Barker et al. 2006). The present results therefore suggest that, within the PRH, there is a single NMDA-dependent synaptic plasticity mechanism, critical for encoding object familiarity, which underlies both novel object recognition memory and object-in-place memory. Whether the same population of neurons is involved in both recognition memory processes, however requires further examination. The present results also demonstrate that the same long-term NMDAR-dependent synaptic plasticity mechanisms in the PRH mediate object-in-place encoding, irrespective of the brain region with which the PRH interacts as previously we have shown that within a PRH-mPFC circuit long-term, but not short-term object-in-place memory depends on NMDAR neurotransmission. Interestingly however, the PRH projections to the HPC and mPFC do not originate from the same population of neurons. Indeed, the direct and indirect (via the lateral entorhinal cortex) input to the HPC arises from the superficial layers (layers I/III) of the PRH (Agster and Burwell 2009) while the input to the mPFC arises preferentially from the deeper layers (Naber et al. 1999); thus, the neurons critical for encoding object memory information appear to be widely distributed through the PRH.

During object-in-place memory, the object familiarity information generated by the PRH must be integrated with the information concerning the location in which the object was encountered. In behavioral tasks that isolate and evaluate different aspects of recognition memory, manipulations of the HPC, PRH, and mPFC have different effects. As the PRH is not critical for spatial memory (Bussey et al. 2000; Barker et al. 2007), it seems unlikely that the object-in-place representation is encoded within the PRH, but rather within the HPC and/or mPFC. Indeed, while a number of studies have shown that lesions mPFC do not impair standard novel object recognition tasks (Mitchell and Laiacona 1998; Barker et al. 2007; Barker and Warburton 2011b), one study has reported impairments following infusions of AP5 into the mPFC (Akirav and Maroun 2006) when 2 different objects were presented in the acquisition phase. While there are additional differences between the studies which found no impairments (lesion studies) and that which did, (intracerebral infusions) the results from the Akirav and Maroun (2006) study are pertinent to the present discussion as they suggest that the PFC is critical for representations of the associative relationships between objects (see also Chao et al. 2013). Furthermore, a recent study showed that neurons in the HPC and PFC represent different types of information during a rule-based object-in-place task. Thus, neuronal firing in the HPC (CA1 region) was found to be spatially localized to represent a specific location while neurons in the prefrontal cortex fired in response to an event, but did not discriminate location (Kim et al. 2011). The hippocampal projection to the mPFC arises in the CA1 subfield and deletion of the NR1subunit of the NMDAR selectively in the CA1, has been shown to result in significant object-place memory impairments (Place et al. 2012). Such findings, together with the results of the present study accord with the hypothesis that a role of the PFC, during object-in-place memory, is to integrate location information from the CA1 subfield of the HPC, with object information from the PRH, using an NMDAR-dependent synaptic plasticity mechanism.

Suggesting a model of object-in-place associative memory in which each region plays a distinct role may, however, be over simplistic. A number of studies have shown that the HPC and prefrontal cortex show coherence in their neuronal activity (Siapas et al. 2005; Kim et al. 2011), and there is evidence of bidirectional control of plasticity between the HPC and PRH (Supcun et al. 2012). Indeed, the fact that, in the present study, inactivating any pair of structures blocked both acquisition and retrieval suggests that object-in-place memory requires network interdependency, rather than the information being held within the neurons of one region (Brown et al. 2012). Therefore, successful object-in-place memory encoding and retrieval appears to be a property of a network of structures which include the HPC, PRH, and mPFC, although dissociations in the effects of NMDAR antagonism reveal that the cellular mechanisms within these different brain regions for the processing of mnemonic information may not need to be identical.

Object-in-place associative recognition memory has been shown to be disrupted in schizophrenic patients (Wood et al. 2002; Burglen et al. 2004) and in animal models of schizophrenia, while single-item recognition is unaffected (Howland et al. 2012). The demonstration that disrupting glutamatergic neurotransmission within a hippocampal-prefrontal system impairs object-in-place memory in rats, accords with these studies. Indeed the HPC and prefrontal cortex and glutamatergic system have each been implicated in the executive dysfunction associated with schizophrenia (for review, see Eisenberg and Berman 2010). Therefore, the results of the present study may be integrated into a wider understanding of the etiology of this condition which provides opportunities to use the link between hippocampal-prefrontal circuitry and object-in-place associative recognition memory to explore the neural basis of cognitive dysfunction in schizophrenia.

## Supplementary Material

Supplementary material can be found at: http://www.cercor.oxfordjournals.org.

## Funding

This work was supported by a project grant from the Biotechnology and Biological Sciences Research Council (grant number: BB/E010407). Funding to pay the Open Access publication charges for this article was provided by The Research Councils UK.

## Supplementary Material

Supplementary Data
