# Supplementary material for: Object-in-Place Associative Recognition Memory Depends on Glutamate Receptor Neurotransmission Within Two Defined Hippocampal-Cortical Circuits: A Critical Role for AMPA and NMDA Receptors in the Hippocampus, Perirhinal, and Prefrontal Cortices
Source: Cereb Cortex. 2013 Sep 12;25(2):472–81. doi: 10.1093/cercor/bht245 (PMC4380082; doi:10.1093/cercor/bht245)
Supplement: Supplementary Data [file supp_25_2_472__index.html]

Object-in-Place Associative Recognition Memory Depends on Glutamate Receptor Neurotransmission Within Two Defined Hippocampal-Cortical Circuits: A Critical Role for AMPA and NMDA Receptors in the Hippocampus, Perirhinal, and Prefrontal Cortices — Object-in-Place Associative Recognition Memory Depends on Glutamate Receptor Neurotransmission Within Two Defined Hippocampal-Cortical Circuits: A Critical Role for AMPA and NMDA Receptors in the Hippocampus, Perirhinal, and Prefrontal Cortices — Supplementary Data 

# Object-in-Place Associative Recognition Memory Depends on Glutamate Receptor Neurotransmission Within Two Defined Hippocampal-Cortical Circuits: A Critical Role for AMPA and NMDA Receptors in the Hippocampus, Perirhinal, and Prefrontal Cortices

## Supplementary Data

Supplementary Data

**Files in this Data Supplement:**

- Supplementary Data - Docx file
- Supplementary Figure 1 - tif file
