# Supplementary material for: Object-in-Place Associative Recognition Memory Depends on Glutamate Receptor Neurotransmission Within Two Defined Hippocampal-Cortical Circuits: A Critical Role for AMPA and NMDA Receptors in the Hippocampus, Perirhinal, and Prefrontal Cortices
Source: Cereb Cortex. 2013 Sep 12;25(2):472–81. doi: 10.1093/cercor/bht245 (PMC4380082; doi:10.1093/cercor/bht245)
Supplement: Supplementary Data [file supp_bht245_bht245supp.docx]

**Supplementary Information**

**Figure Legend for Figure S1**

**Figure S1 No effect of NBQX or AP5 infusions into the HPC-mPFC or HPC-PRH circuits on object recognition and object location memory.** Illustrated for each group is the mean (+sem) discrimination ratio. **a.** Unilateral infusion of NBQX into the HPC-mPFC in opposite hemispheres (HPC-mPFC contra) or the same hemisphere (HPC-mPFC ipsi) had no effect on object recognition or object location tasks(ANOVA treatment by task interaction F(1,16)= 0.75, p>0.1). Further analyses confirmed that NBQX had no effect on the animals ability to discriminate between the novel and familiar objects [HPC-mPFC ipsi t(8)= 15.59, p<0.001; HPC-mPFC contra t(8)= 2.68, p<0.05] or between the objects in the novel or familiar location [HPC-mPFC ipsi t(8)= 5.30, p<0.01; HPC-mPFC contra t(8)= 8.83, p<0.01**]**. **b.** Unilateral infusion of AP5 into the HPC-mPFC in opposite hemispheres (HPC-mPFC contra) or the same hemisphere (HPC-mPFC ipsi) had no effect on object recognition or object location tasks (ANOVA treatment by task interaction F(1,17)= 2.40, p>0.1). Further analyses confirmed that NBQX had no effect on the animals ability to discriminate between the novel and familiar objects [HPC-mPFC ipsi t(10)= 5.74, p<0.001; HPC-mPFC contra t(10)= 10.37, p<0.001] or between the objects in the novel or familiar location [HPC-mPFC ipsi t(7)= 6.40, p<0.001; HPC-mPFC contra t(7)= 3.17, p<0.05**]**. **c.** Unilateral infusion of NBQX into the HPC-PRH in opposite hemispheres (HPC-PRH contra) or the same hemisphere (HPC-PRH ipsi) had no effect on object recognition or object location tasks (ANOVA treatment by task interaction F(1,15)= 1.00, p>0.1). Further analyses confirmed that NBQX had no effect on the animals ability to discriminate between the novel and familiar objects [HPC-PRH ipsi t(7)= 5.45, p<0.01; HPC-PRH contra t(7)= 5.30, p<0.01] or between the objects in the novel or familiar location [HPC-PRH ipsi t(8)= 6.10, p<0.001; HPC-PRH contra t(8)=7.68, p<0.001**]**. **d.** Unilateral infusion of AP5 into the HPC-PRH in opposite hemispheres (HPC-PRH contra) or the same hemisphere (HPC-PRH ipsi) had no effect on object recognition or object location tasks (ANOVA treatment by task interaction (F(1,19)= 0.11, p>0.1). Further analyses confirmed that AP5 had no effect on the animals ability to discriminate between the novel and familiar objects [HPC-PRH ipsi t(9)= 5.83, p<0.001; HPC-PRH contra t(9)= 4.80, p<0.001] or between the objects in the novel or familiar location [HPC-PRH ipsi t(10)= 4.54, p<0.001; HPC-PRH contra t(10)=5.71, p<0.001].
